# Supplementary material for: Spatial and stoichiometric in situ analysis of biomolecular oligomerization at single-protein resolution
Source: Nat Commun. 2025 May 6;16:4202. doi: 10.1038/s41467-025-59500-z (PMC12056017; doi:10.1038/s41467-025-59500-z)
Supplement: Supplementary file 2 — Supplementary Information [file 41467_2025_59500_MOESM2_ESM.pdf]

Supplementary Information

Spatial and stoichiometric *in situ* analysis of biomolecular oligomerization at single-protein resolution

Luciano A. Masullo[1,*,#], Rafal Kowalewski[1,2,*], Monique Honsa[1,2], Larissa Heinze[1,2], Shuhan Xu[1], Philipp R. Steen[1,2], Heinrich Grabmayr[1,2], Isabelle Pachmayr[1,3], Susanne C. M. Reinhardt[1,2], Ana Perovic[1], Jisoo Kwon[1], Ethan P. Oxley[4], Ross A. Dickins[4], Maartje M. C. Bastings[5], Ian A. Parish[6,7], Ralf Jungmann[1,2,#]

[1]Max Planck Institute of Biochemistry, Planegg, Germany.
[2]Faculty of Physics and Center for Nanoscience, Ludwig Maximilian University, Munich, Germany.
[3]Department of Chemistry and Biochemistry, Ludwig Maximilian University, Munich, Germany.
[4]Australian Centre for Blood Diseases, Monash University, Melbourne, Australia.
[5]Institute of Materials and Interfaculty Bioengineering Institute, School of Engineering, École Polytechnique Fédérale de Lausanne, Lausanne, Switzerland.
[6]Cancer Immunology Program, Peter MacCallum Cancer Centre, VIC, Australia.
[7]Sir Peter MacCallum Department of Oncology, The University of Melbourne, VIC, Australia.

*These authors contributed equally to this work. #Correspondence should be addressed to masullo@biochem.mpg.de or jungmann@biochem.mpg.de

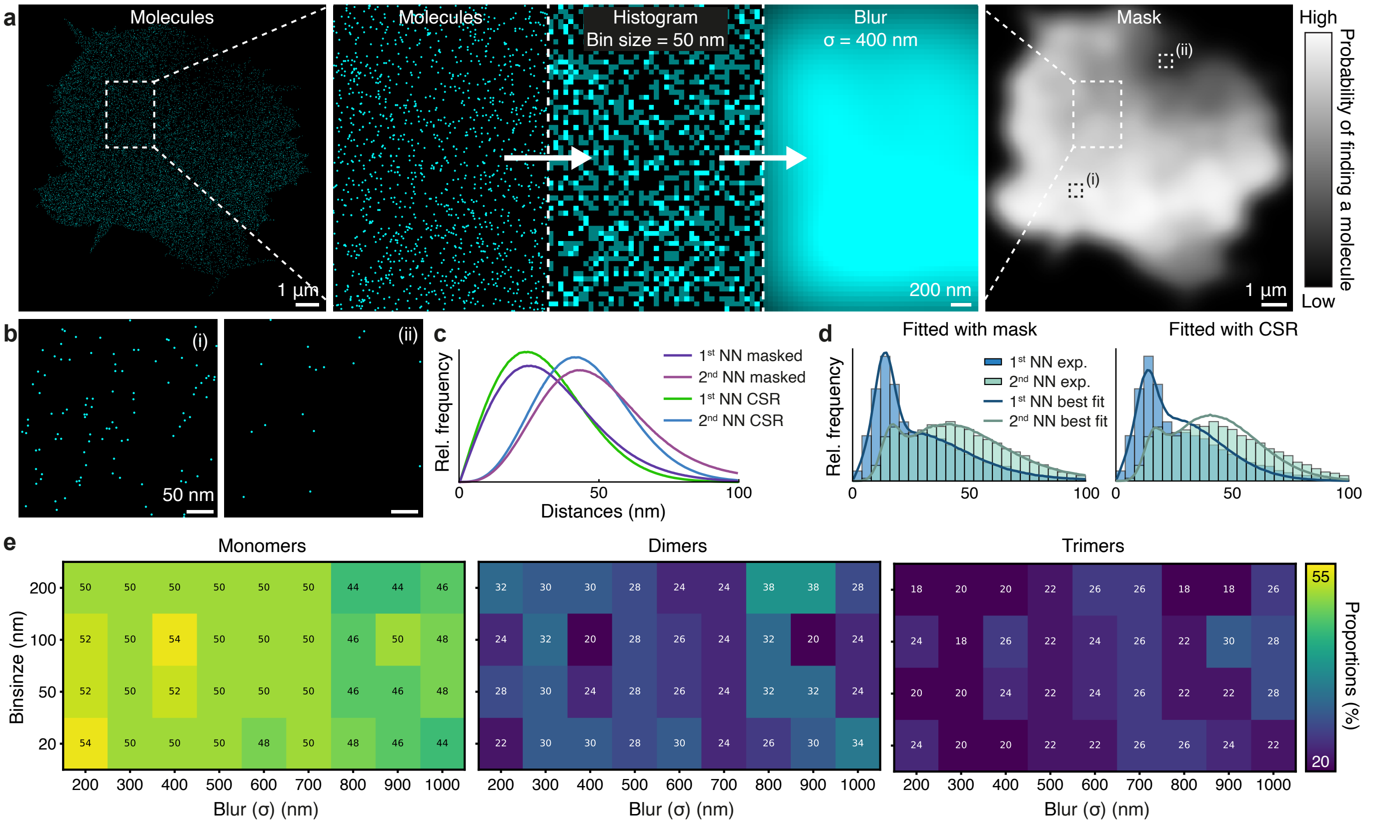

Supplementary Figure 1 | Masking for heterogeneous density recovery. a, Molecules' position (left) can be used to create a mask (right) via histogramming the positions and applying Gaussian blur (middle). Such a mask reflects heterogeneous density across the sample. Each of the pixels in the mask provides the probability of finding a molecule within that pixel. **b,** Two insets from **a** showing resulting molecules simulated using the mask. Inset (i) shows a higher molecular density than inset (ii). 50% monomers, 30% dimers, and 20% trimers were simulated at LE = 50%. **c,** Applying the mask when simulating monomers shows distinct NND distributions compared to the CSR simulation at the average density across the mask. **d,** NND histograms show the ground truth simulated with the mask as in panel **b**. Best SPINNA fit with the mask (left panel) shows a good match (52% monomers, 24% dimers, and 24% tetramers). When CSR is simulated (right panel), a clear deviation is present (72% monomers, 2% dimers, and 26% tetramers). **e,** Recovered stoichiometries across different masking parameters. Recovered proportions of monomers, dimers and trimers are color-coded. The values oscillate around the ground-truth proportions, with maximum deviation of 10 p.p. for a few cases.

Example 1

Structures

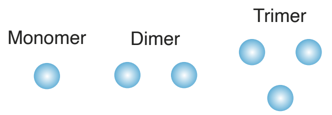

User parameters

Total # of molecules: 1,000
Granularity: 6

Candidate numbers of structures

Possible # of monomers	Possible # of dimers
1000	500
800	400
600	300
400	200
200	100
0	0

6 possible counts

Example resulting mixtures

# of monomers	# of dimers	Resulting # of trimers
800	0	67
800	100	0
400	300	0
0	100	266

Example 2

Structures

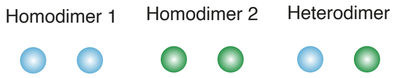

User parameters

Total # of molecules: 20,000
Total # of molecules: 10,000
Granularity: 11

Candidate numbers of structures

Possible # of homodimers 1
10000
9500
9000
8500
8000
7500
7000
6500
6000
5500
5000

11 possible counts

Example resulting mixtures

# of homodimers 1	Resulting # of homodimers 2	Resulting # of heterodimers
10000	5000	0
8000	3000	4500
5000	0	10000

Example 3

Structures

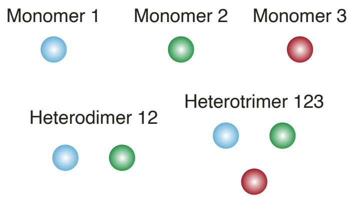

User parameters

Total # of molecules: 1,000
Total # of molecules: 1,000
Total # of molecules: 1,000
Granularity: 5

Candidate numbers of structures

5 possible counts

Possible # of monomers 1	Possible # of monomers 2	Possible # of monomers 3
1000	1000	1000
750	750	750
500	500	500
250	250	250
200	200	200
0	0	0

Example resulting mixtures

# of monomers 1	# of monomers 2	# of monomers 3
500	500	500
0	0	500
250	250	1000

Resulting # of heterodimers 12	Resulting # of heterotrimers 123
0	500
500	500
750	0

Supplementary Figure 2 | Search space generation for stoichiometry fitting. Each column illustrates an example, with model structures and user parameters (total number of simulated molecules of each species and granularity) provided. “Granularity” controls how detailed the search space is, i.e., the higher its value, the finer the differences between the tested stoichiometries are. In the next step, candidate numbers of structures are calculated based on the user parameters. For example, in the left column, given 1,000 molecules, the minimum number of monomers is 0, and the maximum number is 1,000. 6 values within this range are generated. Analogously, 6 values between 0 and 500 are generated for dimers. Lastly, the candidate numbers of structures for each structure are combined in all possible permutations and the numbers of the remaining structures (for example, trimers in the left panel) are calculated such that the total number of simulated molecules corresponds to the user-defined value. See **Supplementary Tables 1, 2 and 3** for the full resulting search spaces of the examples above.

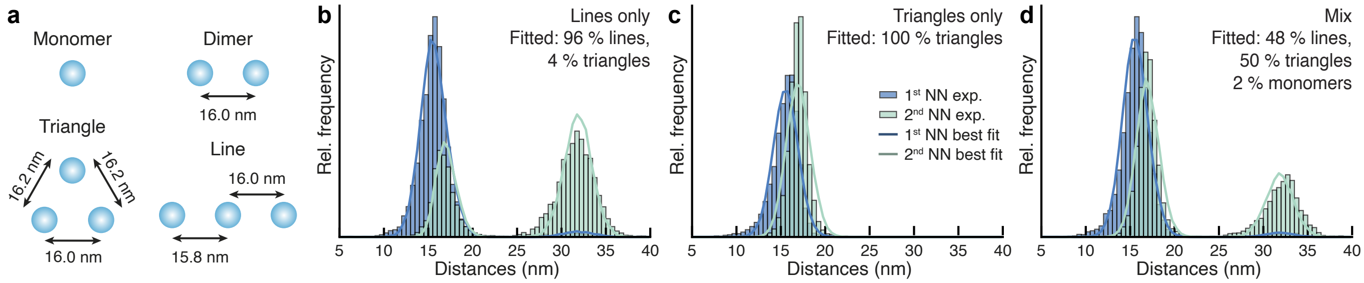

Supplementary Figure 3 | SPINNA on origami datasets with extra structures. a, Model SPINNA structures. Note that monomers and dimers were added to the scenario from Figure 2. **b,** SPINNA results on the lines-only dataset, fitted proportions close to those in Figure 2 were recovered. **c,** SPINNA results on the triangles-only dataset, the same fitted proportions as in Figure 2 were recovered. **d,** Mixed datasets (50 % lines, 50 % triangles). In this case, 48 % lines, 50 % triangles, and 2 % monomers were recovered, close to the result in Figure 2.

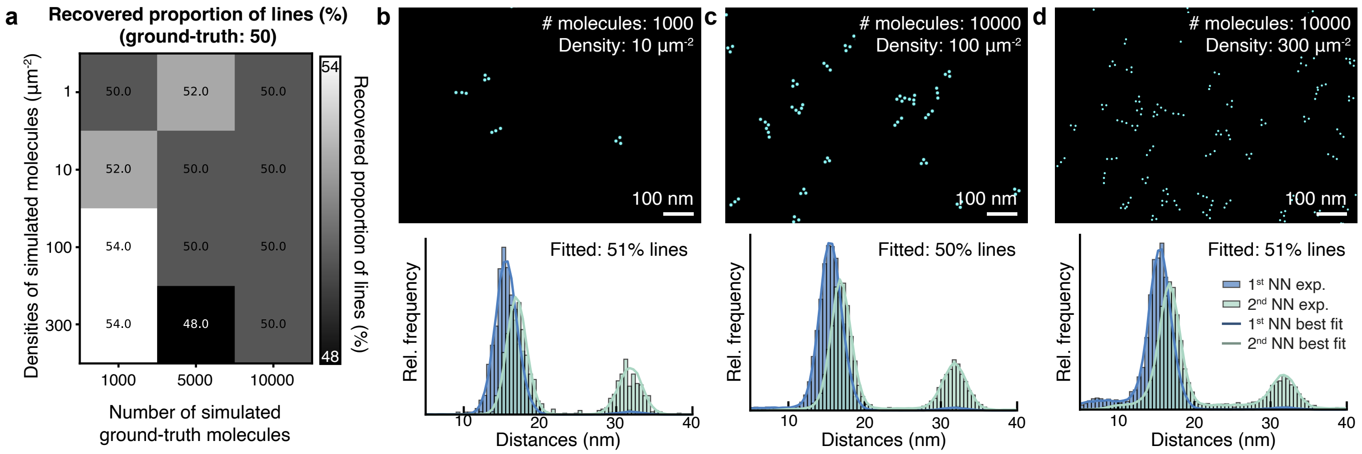

Supplementary Figure 4 | *In silico* validation of origami experiments. **a,** Self-consistency check of SPINNA on the datasets consisting of 50 % of lines and 50 % of triangles across different numbers and densities of simulated molecules. These were fitted using SPINNA and the recovered proportions of lines are reported. **b,** Example simulated data of 1000 molecules at the density of 10 molecules/µm^2 (top) and the resulting NND plot with the best fitting simulation (bottom). 51 % of lines were fitted. **c,** Similar to **b**, except 10,000 molecules were simulated at the density of 100 molecules / µm^2. 50 % of lines were fitted. **d,** Same as **c**, except density of 300 molecules / µm^2 was simulated. Overlaps between structures are present in the simulated data, whose effect is reflected in the NND histograms.

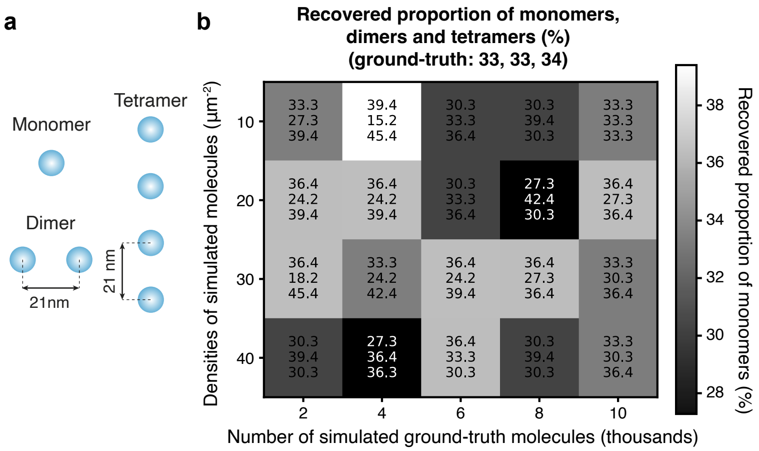

Supplementary Figure 5 | *In silico* validation of SPINNA for the EGFR dataset. a, Model structures used in simulations. **b,** Self-consistency check for the case of simulated ground-truth 33% monomers, 33% dimers, and 34% tetramers. The shade of gray depicts the recovered proportion of monomers using SPINNA, the text shows full recovered stoichiometries.

monomer	dimer	trimer
**1000**	0	0
**800**	0	66
**600**	0	133
**400**	0	199
**200**	0	266
**1**	0	333
**800**	100	0
**600**	100	66
**400**	100	133
**200**	100	199
**0**	100	266
**600**	200	0
**400**	200	66
**200**	200	133
**0**	200	199
**400**	300	0
**200**	300	66
**0**	300	133
**200**	400	0
**0**	400	66
**0**	500	0

Supplementary Table 1 | Full resulting search space of Example 1 in **Supplementary Figure 2**.

homodimer1	homodimer2	heterodimer
**10000**	5000	0
**9500**	4500	1000
**9000**	4000	2000
**8500**	3500	3000
**8000**	3000	4000
**7500**	2500	5000
**7000**	2000	6000
**6500**	1500	7000
**6000**	1000	8000
**5500**	500	9000
**5000**	0	10000

Supplementary Table 2 | Full resulting search space of Example 2 in **Supplementary Figure 2**.

monomer1	monomer2	monomer3	heterodimer12	heterotrimer
**1000**	1000	1000	0	0
**750**	750	750	0	250
**500**	500	500	0	500
**250**	250	250	0	750
**0**	0	0	0	1000
**750**	750	1000	250	0
**500**	500	750	250	250
**250**	250	500	250	500
**0**	0	250	250	750
**500**	500	1000	500	0
**250**	250	750	500	250
**0**	0	500	500	500
**250**	250	1000	750	0
**0**	0	750	750	250
**0**	0	1000	1000	0

Supplementary Table 3 | Full resulting search space of Example 3 in **Supplementary Figure 2**.

Supplementary Data legends

Supplementary Data 1 | SPINNA results in Figure 3. Sheets show the recovered stoichiometries in panels g, h and i of Figure 3. Additionally, the impact of uncertainty in LE is investigated in a separate sheet by varying the values of LE input (mean LE +/- st. dev, LE) in SPINNA for each cell.

Supplementary Data 2 | SPINNA results in Figure 4. Sheets show the recovered stoichiometries in panels d, e and f of Figure 4. Additionally, the impact of uncertainty in LE is investigated in separate sheets by varying the values of LE input (mean LE +/- st. dev, LE) in SPINNA for each cell.

Supplementary Data 3 | Scaffold sequence (M13mp18) of DNA origami used in Figure 2.

Supplementary Data 4 | Staple-strand sequences of DNA origami used in Figure 2.
